# Iron deficiency in children and adolescents with attention deficit hyperactivity disorder: Does the relationship exist?

**DOI:** 10.4102/sajpsychiatry.v24i0.1284

**Published:** 2018-09-20

**Authors:** Luzuko Magula, Karis Moxley, Anusha Lachman

**Affiliations:** 1 Department of Psychiatry, Faculty of Medicine and Health Sciences, Stellenbosch University, South Africa

## Abstract

**Background:**

Iron is a cofactor in the production and breakdown of neurotransmitters like dopamine and is vital for normal brain function. Iron deficiency potentially contributes to the development of attention deficit hyperactivity disorder (ADHD) because dopamine imbalances can cause hyperactivity, restlessness and problems with concentration and attention. However, a direct association between iron deficiency and ADHD remains to be determined.

**Objectives:**

The aim of this study was to investigate the possible correlation between iron deficiency and ADHD in children and adolescents seen at the child psychiatry outpatient service at Tygerberg Hospital, South Africa.

**Methods:**

A retrospective chart review was conducted to gather data of all outpatient children and adolescents who had their serum ferritin and/or iron levels tested between February 2011 and January 2016. Relevant demographic and clinical information was extracted from 255 records, and statistical methods were used to test for correlations between ADHD and certain variables, including iron deficiency.

**Results:**

Out of 255 patients, 88 (34.5%) had iron deficiency, 157 (61.6%) had ADHD and 54 (21.0%) had both iron deficiency and ADHD. Of those patients with ADHD, 11 (7.0%) had other psychiatric comorbidities, and more males (89.0%) had this dual diagnosis compared to females (11.0%). Variables found to be significantly associated with ADHD included gender, age, Ritalin treatment and psychiatric comorbidities, but there was no significant association between ADHD and iron deficiency (*p* = 0.150).

**Conclusion:**

There was no relationship between ADHD and iron deficiency in this cohort of children and adolescents. Further studies using a treatment-naïve sample are required.

